# Characterization of Urban Runoff Pollution between Dissolved and Particulate Phases

**DOI:** 10.1155/2013/964737

**Published:** 2013-07-11

**Authors:** Zhang Wei, Li Simin, Tang Fengbing

**Affiliations:** School of Urban Construction, Hebei University of Engineering, Handan, Hebei 056038, China

## Abstract

To develop urban stormwater management effectively, characterization of urban runoff pollution between dissolved and particulate phases was studied by 12 rainfall events monitored for five typical urban catchments. The average event mean concentration (AEMC) of runoff pollutants in different phases was evaluated. The AEMC values of runoff pollutants in different phases from urban roads were higher than the ones from urban roofs. The proportions of total dissolved solids, total dissolved nitrogen, and total dissolved phosphorus in total ones for all the catchments were 26.19%–30.91%, 83.29%–90.51%, and 61.54–68.09%, respectively. During rainfall events, the pollutant concentration at the initial stage of rainfall was high and then sharply decreased to a low value. Affected by catchments characterization and rainfall distribution, the highest concentration of road pollutants might appear in the later period of rainfall. Strong correlations were also found among runoffs pollutants in different phases. Total suspended solid could be considered as a surrogate for particulate matters in both road and roof runoff, while dissolved chemical oxygen demand could be regarded as a surrogate for dissolved matters in roof runoff.

## 1. Introduction

With rapid urbanization and industrialization, surface water quality of urban watersheds has deteriorated gradually in many cities of the world. To tackle this problem, much attention has been paid to reducing pollutant loads of point sources. However, the water quality has not been improved obviously [1]. Urban runoff has been considered as one of the primary causes of water quality degradation [2, 3]. And along with the expansion of point sources control, the contribution of water quality degradation from urban runoff pollution is increasing.

Understanding the characteristics of urban runoff pollution is beneficial to develop urban stormwater management effectively. Due to the randomicity of natural rainfall and the complexity of urban catchments, urban runoff pollution is characterized by the occurrence of great temporal and spatial variability [4]. Urban runoff pollutants are divided into six specific groups, such as solids, heavy metals, biodegradable organic matter, organic micropollutants, pathogenic microorganisms, and nutrients, which are originated mainly from wet and dry deposition, grind tire debris, vegetation (leaves and logs), animals (fecal contributions and dead bodies), fertilizers, and exhaust gas from vehicle, and so forth [5, 6]. In many literatures on urban runoff pollution, the analyses of measurement parameters were usually carried out on whole water samples [7–9]. Furthermore, some researchers deem that particle substances are the main category of urban runoff pollutants, while particle size distribution on impervious surfaces in urban environments determines the characteristics of urban runoff pollution [10–12].

Actually, runoff pollutants in the environment exist mainly in the form of particulate and dissolved phase, which is one of leading factors on selection of stormwater quality control measures [13–15]. However, little information is available on characterizing the dissolved pollutants in urban runoff, especially the comparison of runoff pollutants between particulate and dissolved phase. The main objective of this study focuses on characterizing the discharge of runoff pollutants in different phases from urban typical catchments.

## 2. Materials and Methods

### 2.1. Study Sites

Handan city is located in the south portion of Hebei province, China, which is geographically located between north latitude 36°20′–36°44′ and east longitude 114°03′–114°40′. In Handan city, the mean annual rainfall is 558.5 mm and the average annual temperature is 13.5°C. Urban district covered an area of 118.6 km^2^ in 2009, and its population was 1.16 million. For this study, two roads and three roofs were selected as urban typical catchments. The catchments characteristics were shown in Table 1.

### 2.2. Monitoring and Sampling

In this study, 12 rainfall events were collected in 2011. Rainfall data were monitored by a telemetry rain gauge (SL1, China), which was placed near all the study sites.  Table 2 provided the dates of monitoring events and related parameters.

The sample collection was performed simultaneously in five study sites. Road runoff was sampled at stormwater inlets, while roof runoff was at the bottom of the vertical drain pipes. At the beginning of runoff generation, samples were collected at every 5 minutes intervals. When the rainfall duration was more than 30 minutes, sampling interval would be extended to 10–30 minutes until the runoff disappeared or water quality became gradually stable [16]. More than 9 samples were taken during a rain event.

### 2.3. Data Analyses

The simples of both road and roof runoff were tested for total solids (TS), total dissolved solids (TDS), total chemical oxygen demand (TCOD), dissolved chemical oxygen demand (DCOD), total nitrogen (TN), dissolved total nitrogen (DTN), total phosphorus (TP), and dissolved total phosphorus (DTP). Unfiltered samples were analyzed for TS, TCOD, TN, and TP, which represent total pollutants of urban runoff. For determination of dissolved pollutants represented by TDS, DCOD, DTN, and DTP, the samples were pretreated by a 0.45 *μ*m Millipore filter membrane [17, 18]. All parameters were analyzed in accordance with standard methods specified in APHA 2005 [19]. Moreover, the concentration of particulate pollutants which included total suspended solid (TSS), particulate chemical oxygen demand (PCOD), particulate total nitrogen (PTN), and particulate total phosphorus (PTP) could be determined by total ones minus dissolved ones [20].

## 3. Results and Discussion

### 3.1. AEMC of Runoff Pollutants from Different Catchments

Event mean concentration (EMC) has been widely used to evaluate the runoff pollution load for receiving waters in an individual storm event [21], which is given by the following equation:

(1) $$\begin{array}{c} \text{EMC}=\frac{\int_{0}^{T}Q_{t}C_{t}\text{d}t}{\int_{0}^{T}Q_{t}\text{d}t}\approx \frac{\sum_{0}^{T}{\overset{-}{Q}}_{t}{\overset{-}{C}}_{t}\Delta t}{\sum_{0}^{T}{\overset{-}{Q}}_{t}\Delta t}, \end{array}$$

where *T* is rainfall duration; Δ*t* is time interval of sampling; ${\overset{-}{Q}}_{t}$,  ${\overset{-}{C}}_{t}$ are mean runoff quantity and pollutant concentration at time interval, respectively.

 However, affected by rainfall distribution and catchments types, EMC of urban runoff is significantly variable in each event or in each catchment [22]. Then, average EMC (AEMC) was put forward to predict the overall runoff quality accurately by data from more than one rainfall event [23]. Based on statistical analysis for a total of 645 samples during 12 rainfall events, the AEMC values of runoff pollutants in different phases were shown in Table 3. 

In general, the AEMC values of runoff pollutants in different phases from urban roads (RD1 and RD2) were higher than the ones from urban roofs (RF1, RF2, and RF3). It was suggested that road runoff is more polluted than roof runoff. Compared with RD2, runoff pollution of RD1 was more serious because of heavier traffic. Pollution rank of roof runoff was in the following order: RF1 > RF2 > RF3. It might be caused that the cover material of RF1 is asphalt, which was easy to age and chip after a long time use under the outdoor environment. And low AEMC of RF3 was attributed to using tile as cover material to a certain extent, because the production of some pollutants would be reduced by the good erosion-corrosion resistance of tile roof during the runoff process. 

Furthermore, distributions of runoff pollutants for all the catchments were similar except for DCOD. The proportions of TDS, DTN, and DTP in total ones were 26.19%–30.91%, 83.29%–90.51%, and 61.54%–68.09%, respectively. It could be observed that solids exist as particulate phase in urban runoff. Conversely, dissolved matters are the mainly existing phase of nutrients which is usually expressed as nitrogen and phosphorus [24]. Based on the monitoring data, distribution of organic pollutants was closely related to the cover material of urban catchments. For asphalt road and roof (RD1, RD2, and RF1), the proportions of DCOD in TCOD were in the range from 71.76% to 86.23%. But because about half TCOD exist as dissolved phase, the distribution of organic pollutants was equally represented in rainfall runoff from the catchments used inorganic cover material, such as concrete and tile.

### 3.2. Characterizing Runoff Pollutants in Different Phases during Typical Rainfall Event

**Figure 1:**
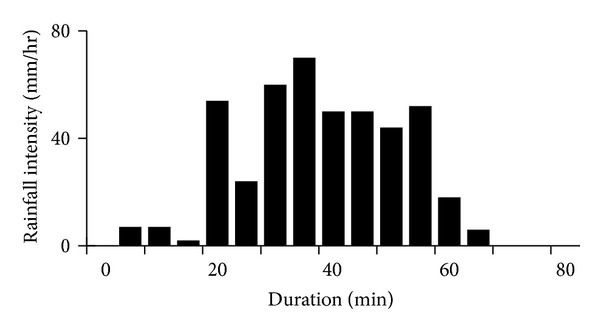
Distribution of typical rainfall (July 20, 2011).

During a rainfall event, pollutants transport in different phases is affected by several factors, such as rainfall distribution, catchments type, and pollutant component. Taking a rainfall event of July 20, 2011 (22.2 mm) for example, the rainfall distribution was showed in Figure 1. 

**Figure 2:**
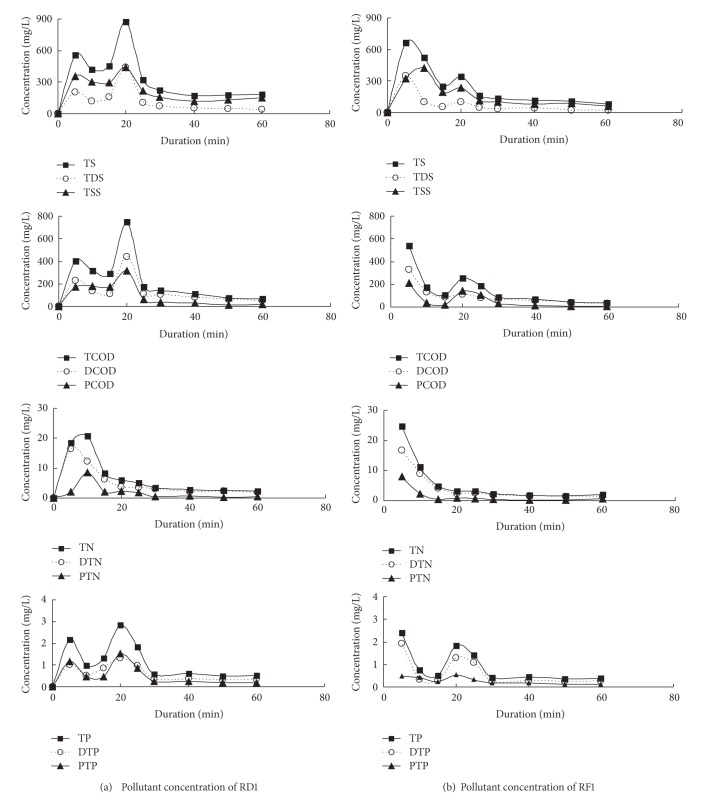
Runoff pollutograph from RD1 and RF1 during rainfall event of July 20, 2011.

Figure 2 showed the variation of pollutant concentrations in different phases. Most runoff pollutants from both RD1 and RF1 were mostly characterized by the tendency that the concentration at the initial stage of rainfall was high and then sharply decreased to a low value. As showed in Table 1, the high intensity rainfall occurred in the later period, so pollutant concentrations increased in various degrees. The highest concentration of RD1 (except nutrients) appeared in the later period of rainfall when the rainfall intensity rose dramatically, whereas that of RF1 occurred at the beginning of runoff generation. It was possible that the washoff of runoff pollutants in RF1 was less suffered from the influence of rainfall intensity due to its small area and smooth surface. However, for the road (RD1), some pollutants, especially particulate matter, might be intercepted by coarse surface during low intensity rainfall, which could not be washed off into the runoff until rainfall intensity rose to reach sufficient strength. The concentrations of nutrients, including TN, DTN, and PTN, have little or no effect by the variety of rainfall intensity. The reason might be that nitrogen in water environment was found mainly in four forms: ammonia, nitrite, nitrate, and organic nitrogen. Only part of organic nitrogen exists as particulate phase, and others are all dissolved matter [25]. The characterization of previous runoff pollutants also reappeared for other rainfall events monitored in this study.

### 3.3. Correlation Analysis between Runoff Pollutants in Different Phases

According to previous research, there were good linear correlations between runoff pollutants. Several pollutants could be considered as surrogates for others so as to reduce the enormous costs to monitor [26–28]. In this study, correlations of runoff pollutants were performed very closely at the same kinds of catchments, so linear correlations of runoff pollutants in different phases were analyzed using combined data, in which data of roof pollutants was collected from RF1, RF2, and RF3, and that of road pollutants was from RD1 and RD2. Pearson's coefficient (*r*) was used for ranking the correlation. When *r* ≥ 0.8, it could be determined as strong correlation between runoff pollutants [29]. Correlation analysis results between runoff pollutants in different phases from roof and road were showed in Table 4.

Among both road and roof runoff pollutants, TSS showed a strong correlation with particulate matters, except for PTN. It might be caused that there were significant differences between PTN and other pollutants of influencing factor on concentration variation mentioned earlier. And one pollutant in different phases was also strongly correlated with each other exclusive of PCOD-DCOD, TSS-TDS in road runoff, and TSS-TDS in roof runoff. Besides, DCOD was strongly correlated with dissolved matters in roof runoff; however, similar results were not found in road runoff. Therefore, TSS could be considered as a surrogate for particulate matters in both road and roof runoffs, while DCOD could be regarded as a surrogate for dissolved matters in roof runoff. 

## 4. Conclusions

This paper focused on characterization of urban runoff pollution between dissolved and particulate phases. The results showed that the AEMC values of runoff pollutants in different phases from urban roads were higher than the ones from urban roofs. The proportions of total dissolved solids, dissolved total nitrogen, and dissolved total phosphorus in total ones for all the catchments were 26.19%–30.91%, 83.29%–90.51%, and 61.54%–68.09%, respectively. During rainfall events, the concentration at the initial stage of rainfall was high and then sharply decreased to a low value. Affected by catchments characterization and rainfall distribution, the highest concentration of road pollutants might appear in the later period of rainfall. Strong correlations were also found among runoff pollutants in different phases. Among both road and roof runoff pollutants, TSS shows a strong correlation with particulate matters, except for PTN. DCOD is strongly correlated with dissolved matters in roof runoff.

## Figures and Tables

**Table 1:** Characteristics of urban typical catchments.

Study site	Area (m^2^)	Cover material	Gradient (%)	Traffic flow (vehicles/h)	Cleaning frequency
Road 1, RD1	900	Asphalt	2.0	1440	Once a day
Road 2, RD2	240	Asphalt	1.5–2.0	400	Once a day
Roof 1, RF1	90	Asphalt	2.5	—	—
Roof 2, RF2	140	Concrete	3.0	—	—
Roof 3, RF3	120	Tile	100	—	—

—: no person activity and no cleaning.

**Table 2:** Rainfall dates and related parameters of monitoring events in this study.

No.	Event date (mm/dd)	Total rainfall (mm)	Rainfall duration (min)	Average rainfall intensity (mm/hr)	Antecedent dry day (days)
1	05/20	5.5	162	2.0	11
2	06/07	2.5	86	1.7	17
3	06/24	10.3	460	1.3	16
4	07/02	17.9	295	3.6	7
5	07/20	22.2	65	20.5	16
6	07/29	58.2	270	12.9	8
7	08/01	26.4	660	2.4	1
8	08/16	10.7	60	10.7	5
9	09/11	32.7	1140	1.7	1
10	09/14	7.0	420	1.0	2
11	09/16	32.9	630	3.1	2
12	10/10	3.1	140	1.3	2

**Table 3:** AEMC values and proportion of runoff pollutants in different phases.

Parameters*	Study sites				
	RD1**	RD2**	RF1**	RF2**	RF3**
TS	220	185	168	172	138
TDS	68	49	44	51	40
%***	30.91	26.49	26.19	29.65	28.99
TCOD	119.98	113.80	90.15	75.56	53.72
DCOD	86.23	75.01	71.76	34.99	26.42
%***	71.87	65.91	79.60	46.31	49.18
TN	4.27	4.11	3.89	3.15	3.21
DTN	3.86	3.72	3.24	2.68	2.79
%***	90.40	90.51	83.29	85.08	86.92
TP	0.67	0.58	0.47	0.32	0.26
DTP	0.45	0.38	0.32	0.21	0.16
%***	67.16	65.52	68.09	65.63	61.54

*All the parameters units are mg/L except for “%.”

**RD1: road 1; RD2: road 2; RF1: roof 1; RF2: roof 2; RF3: roof 3.

***Proportion of dissolved ones in total ones.

**Table 4:** Correlation between runoff pollutants in different phases.

Correlation matrix												
	TS	TDS	TSS	TCOD	DCOD	PCOD	TN	DTN	PTN	TP	DTP	PTP
TS		**0.976**	**0.972**	**0.830**	0.651	**0.870**	0.425	0.414	0.331	**0.914**	0.489	**0.914**
TDS	**0.886**		0.689	**0.884**	0.583	0.721	0.242	0.239	0.181	**0.898**	0.565	**0.805**
TSS	**0.925**	0.644		**0.842**	0.565	**0.872**	0.504	0.584	0.478	**0.882**	0.469	**0.874**
TCOD	0.761	0.668	0.629		**0.874**	**0.865**	0.394	0.369	0.338	0.679	0.639	0.723
DCOD	0.711	**0.893**	0.691	**0.918**		0.781	0.257	0.260	0.183	0.584	0.522	0.714
PCOD	0.720	0.549	0.894	**0.849**	**0.838**		0.528	0.472	0.496	0.616	0.703	0.610
TN	0.514	0.463	0.421	0.625	**0.873**	0.716		**0.957**	**0.814**	0.332	0.301	0.349
DTN	0.431	0.540	0.367	0.569	**0.859**	0.677	**0.996**		**0.830**	0.372	0.339	0.390
PTN	0.557	0.783	0.309	0.624	0.576	0.775	**0.982**	**0.960**		0.161	0.143	0.171
TP	0.725	0.637	0.511	0.640	0.726	0.695	0.704	0.668	0.759		**0.985**	**0.991**
DTP	0.674	0.639	0.425	0.635	**0.821**	0.593	0.698	0.654	0.770	**0.993**		**0.955**
PTP	**0.820**	0.704	**0.858**	0.416	0.424	**0.857**	0.618	0.620	0.599	**0.877**	**0.813**	

The data in the table are all the correlation coefficient “*r*.”

The data of correlation coefficient between road runoff pollutants is above the diagonal; in contrast, that between roof runoff pollutants is below the diagonal.

High correlation coefficients (*r* ≥ 0.8) are shown in bold.
